# Invasive *Aspergillus versicolor* infection of the oral and vaginal mucosa in a patient with Lichen planus

**DOI:** 10.22034/cmm.2025.345382.1638

**Published:** 2026-02-01

**Authors:** Dima Ibrahim, Fatima AlDhaheri, Akela Ghazawi, Seema Oommen

**Affiliations:** 1 Department of Laboratory, Burjeel Medical City, Abu Dhabi, UAE; 2 College of Medicine and Health Sciences, Khalifa University, Abu Dhabi, UAE; 3 College of Medicine and Health Sciences, United Arab Emirates University, Abu Dhabi, UAE

**Keywords:** Aspergillosis, *Aspergillus versicolor*, Lichen planus, Mucosal infection

## Abstract

**Background and Purpose:**

Invasive aspergillus stomatitis and vaginitis are relatively rare in a patient with Oral Lichen planus and a history of steroid and antibiotic use.

**Case Presentation:**

A 49-year-old female diagnosed with lichen planus presented with severe stomatitis and cheilitis and was treated with fluconazole as the clinical appearance resembled thrush. Recurrence of her symptoms despite adequate treatment led to culture from the lesions, which repeatedly grew *Aspergillus* spp., an unlikely pathogen at this site. Sequencing further identified the isolate as *Aspergillus versicolor*. Antifungal treatment was initiated, first with micafungin and then with voriconazole. The patient responded and was discharged, only to return with a relapse of symptoms. Environmental samples from her house revealed the same *Aspergillus* spp. The patient responded to isavuconazole and was discharged home after thorough house decontamination, with no recurrence of lesions on follow-up.

**Conclusion:**

Aspergillosis should be considered a differential diagnosis in mucosal lesions that are difficult to treat in both immunocompetent as well as immunocompromised individuals.

## Introduction

Oral lichen planus (OLP) is an inflammatory disorder that affects mainly the skin and mucous membranes of the oral cavity [1]. It is a T-cell-mediated disease-causing apoptosis of basal cells [2]. It can be triggered by genetic dysfunction and environmental factors [1]. Many studies have mentioned an association between OLP and oral fungal infection, especially candidiasis. Treatment with steroids, either topical or systemic, was associated with a higher incidence of candidiasis of the oral mucosa in patients with OLP [3].

Literature describes mold fungal infection of the oral mucosa more commonly in immunocompromised individuals, like hematologic malignancy patients, than in immunocompetent individuals [4]. Yoshinari Myoken et al. described 12 cases of invasive aspergillus stomatitis in patients with acute leukemia [4]. In 1997, Leonard et al. reported the findings of an autopsy of the oral mucosa of 20 acquired immunodeficiency syndrome patients [5]. Oral invasive aspergillosis in a non-immunocompromised patient is extremely rare. This study aimed to report an unusual case of invasive aspergillus stomatitis and vaginitis in an otherwise immunocompetent patient with OLP with a background of steroid and antibiotic use.

## Case Presentation

This study reports the case of a 49-year-old female with diabetes (well controlled), diagnosed as having Lichen Planus of the oral mucosa since June 2022, and treated with topical steroids. She presented initially to the Infectious Diseases clinic on 4 January 2023 for severe oral stomatitis and cheilitis that started after intake of multiple antibiotic courses for genital abscesses. Clinical examination was consistent with severe thrush. She was not responding clinically well to oral fluconazole and a short course of fluconazole (intramuscular injection) that was prescribed in the internal medicine clinic. She was admitted and started on fluconazole 400 mg (intravenous injection) daily with progressive improvement. Dermatology consultation suspected exacerbation of lichen planus, and she was started on prednisolone 60 mg, to be tapered over 10 days. She was discharged on 9 January 2023 on oral fluconazole 200 mg daily for an additional eight days (to complete a total 14-day course) as well as to taper prednisone.

She returned to the Infectious Diseases clinic on 20 January 2023 for recurrence of oral pain and a whitish tongue. Therefore, 200 mg of oral fluconazole was initiated; however, her symptoms continued to worsen. She was shifted to oral itraconazole solution (200 mg) daily due to concern for fluconazole-resistant *Candida* resulting from prior exposure.

She returned to the clinic on 25 January 2023 with worsening, painful oral mucositis. She was admitted, and a culture was obtained from the tongue to assess antifungal resistance in *Candida* spp. A KOH mount was not requested as the clinical suspicion was drug-resistant candidiasis. Micafungin 100 mg IV daily for 2 days was started and was increased to 150 mg IV daily due to very slow improvement. Upper and lower endoscopies were performed and revealed findings consistent with fungal esophagitis (Figure 1).

**Figure 1:**
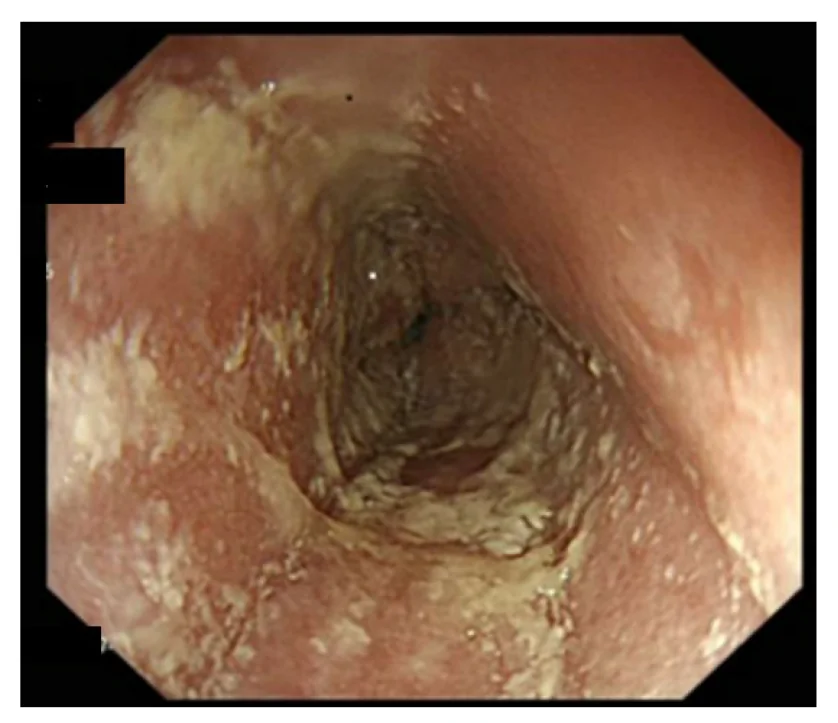
Upper gastrointestinal endoscopy showing grade 3 fungal esophagitis

The fungal cultures were grown on Sabouraud’s Dextrose agar with and without antibiotics, and incubated at 30 °C. Cultures initially grew pure, flat, white colonies within three to four days, morphologically resembling *Candida* spp. Over the course of the week, the colonies slowly developed a velvety texture but remained white in color (Figure 2A). Microscopic examination of the colonies was surprisingly consistent with *Aspergillus* species (Figure 2C). By the second week, colonies developed a green color with a pinkish border (Figure 2B). It was decided to sequence the isolate for confirmation since it was obtained from an uncommon site for this pathogen; the colony morphology was not exactly consistent with the usual *Aspergillus* spp. by the first week, and treatment options differ between aspergillosis and candidiasis. Sanger sequencing was performed using the Applied Biosystems 3500 Genetic Analyzer with internal transcribed spacer, β-tubulin, and Calmodulin regions for confirmation. Sequencing confirmed the diagnosis as *Aspergillus* spp., with the final identification as *Aspergillus versicolor*. Antifungal susceptibility testing of molds is not presently available in our laboratory or anywhere else in the country. *Aspergillus* galactomannan was measured in blood and found to be 1.04, with a cutoff for positivity of 0.5.

**Figure 2:**
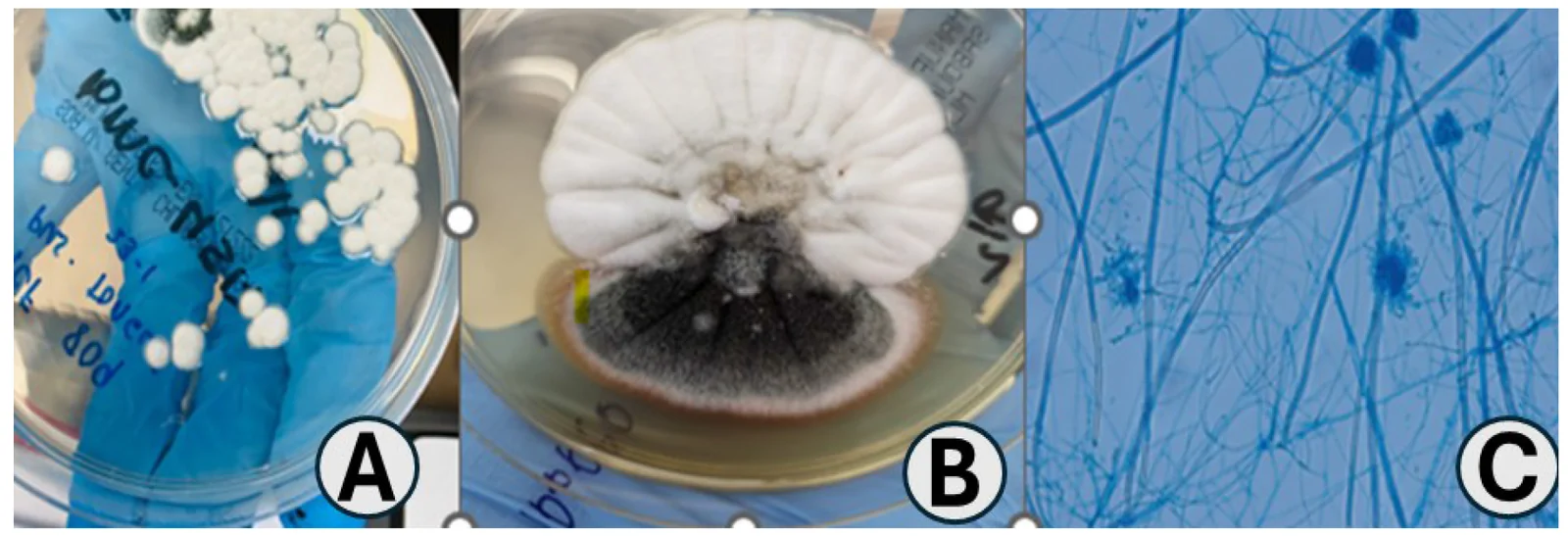
Fungal colony morphology at the end of the first (A) and second week (B), as well as microscopic morphology (C)

During her hospital stay, a tongue biopsy was performed on January 30 and revealed erosive lichen planus. Moreover, no fungal elements were observed on either Haematoxylin and Eosin or Gomori Methenamine stains. This could be explained as she was on antifungal therapy. During her admission, she improved significantly on IV micafungin with no steroid treatment. Due to positive culture for *Aspergillus* spp. and improvement on high-dose micafungin, the patient was discharged on February 4 with oral voriconazole rounded to 300 mg twice daily (4 mg/kg orally) following inpatient loading with 6 mg/kg IV twice daily.

She was followed in the clinic with stabilization of oral lesions. Voriconazole dose was decreased to 200 mg twice daily due to significant disturbance of liver function tests. The voriconazole level was measured at 5.6 µg/mL. Shortly after decreasing the dose, the patient developed worsening of oral lesions along with odynophagia. Voriconazole was discontinued, and oral isavuconazole was initiated at a dose of 200 mg daily on 17 February 2023.

The patient returned one week later with stable but slowly improving oral lesions and vaginal discharge. She was re-admitted, and cultures from the oral mucosa and vagina were positive again for *Aspergillus versicolor*. She was transitioned to IV isavuconazole with significant improvement of symptoms observed within 3-4 days. After one week of treatment, she was discharged on oral isavuconazole; however, she re-presented for recurrence of her symptoms on 20 March 2023.

She was readmitted on 21 March 2023 and was transitioned from oral to IV isavuconazole with clinical improvement in 3-4 days. She was given a total of 24 days of antifungal IV in the hospital, followed by 34 days of IV treatment on an outpatient basis. Figure 3 illustrates the progressive resolution of oral lesions while on IV isavuconazole.

**Figure 3:**
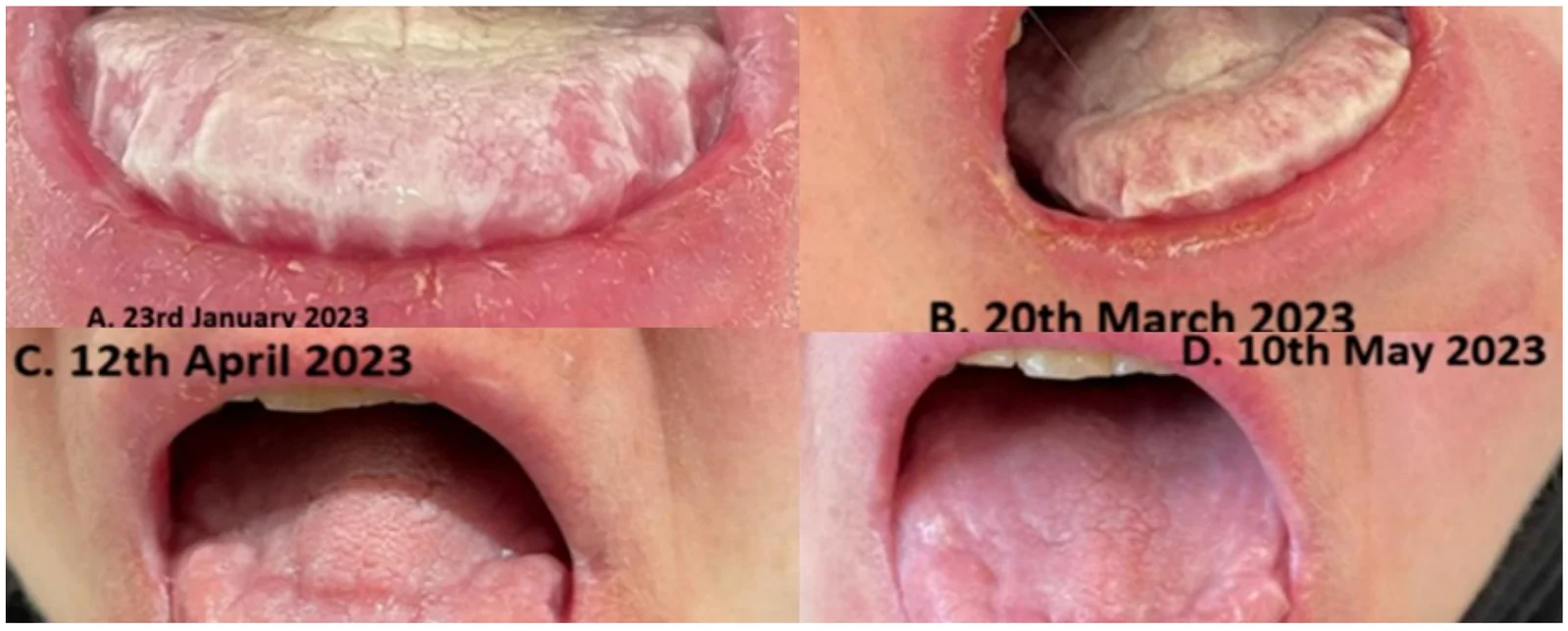
Progressive resolution of oral mucosal lesions

Immunodeficiency was investigated, and it was found that human immunodeficiency virus antibodies were negative, and a subset of immunoglobulin levels were normal (IgG: 8.85 g/l, IgM: 1.8 g/l, IgA: 2.87 g/l, IgE: 4 U/ml). Peripheral blood flow cytometry for T cells, B cells, and NK cells, including absolute CD3, CD4, and CD8 counts, and the CD4/CD8 ratio was negative. Computed tomography of the chest and sinus showed no radiological evidence of invasive aspergillosis. Repeat upper gastrointestinal endoscopy during her last hospital stay (Figure 4) showed no signs of esophageal fungal infection. Esophageal biopsy was consistent with extensive acute on top of chronic esophagitis with dyskeratosis and thickening of the basement membrane, suggestive of involvement by a known systemic lichenoid inflammatory disorder.

**Figure 4:**
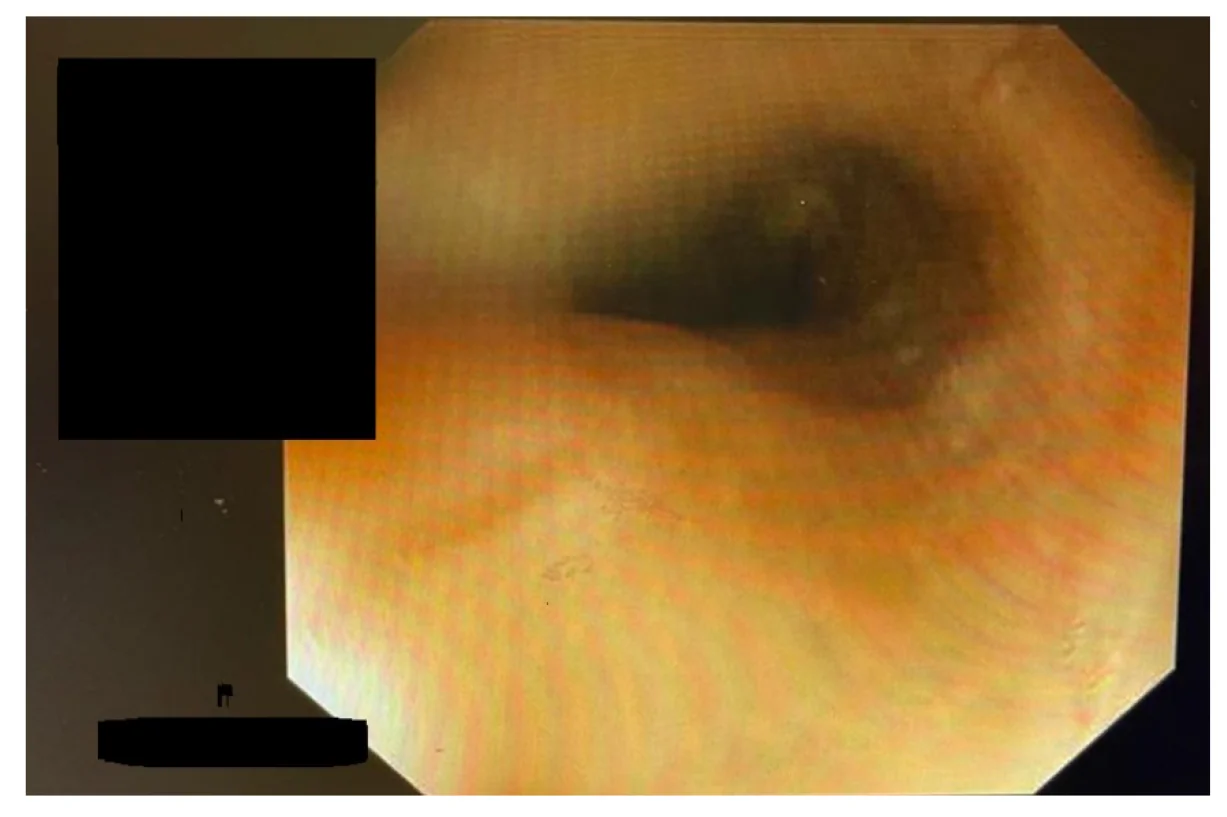
Repeat upper gastrointestinal endoscopy during her last hospital stay, showing resolution

Given recurrent flare after discharge to home, various environmental sites were cultured on Sabouraud’s dextrose agar incubated at 30 °C for 14 days. *Aspergillus versicolor* (identified morphologically and by matrix-assisted laser desorption ionization–time of flight mass spectrometry [MALDI-TOF]) was isolated from the air conditioning vent swab culture. During her hospital stay, fumigation and deep cleaning were performed at home, including the air conditioner and its vents.

In early May 2023, the dermatology team initiated cyclosporin for erosive lichen planus while showing no clinical signs of active fungal infection, but she was maintained on oral isavuconazole. Clinical follow-up showed complete resolution of the oral mucositis with improvement of vaginal discharge and perineal erythema Isavuconazole was stopped on 17 July 2023. After that, the patient had a few episodes of worsening oral pain that was attributed by the dermatologist to Lichen planus and improved after starting immunosuppression (steroids/cyclosporin). After that date, there was no evidence of active fungal infection. Figure 5 illustrates the timeline of events.

**Figure 5:**
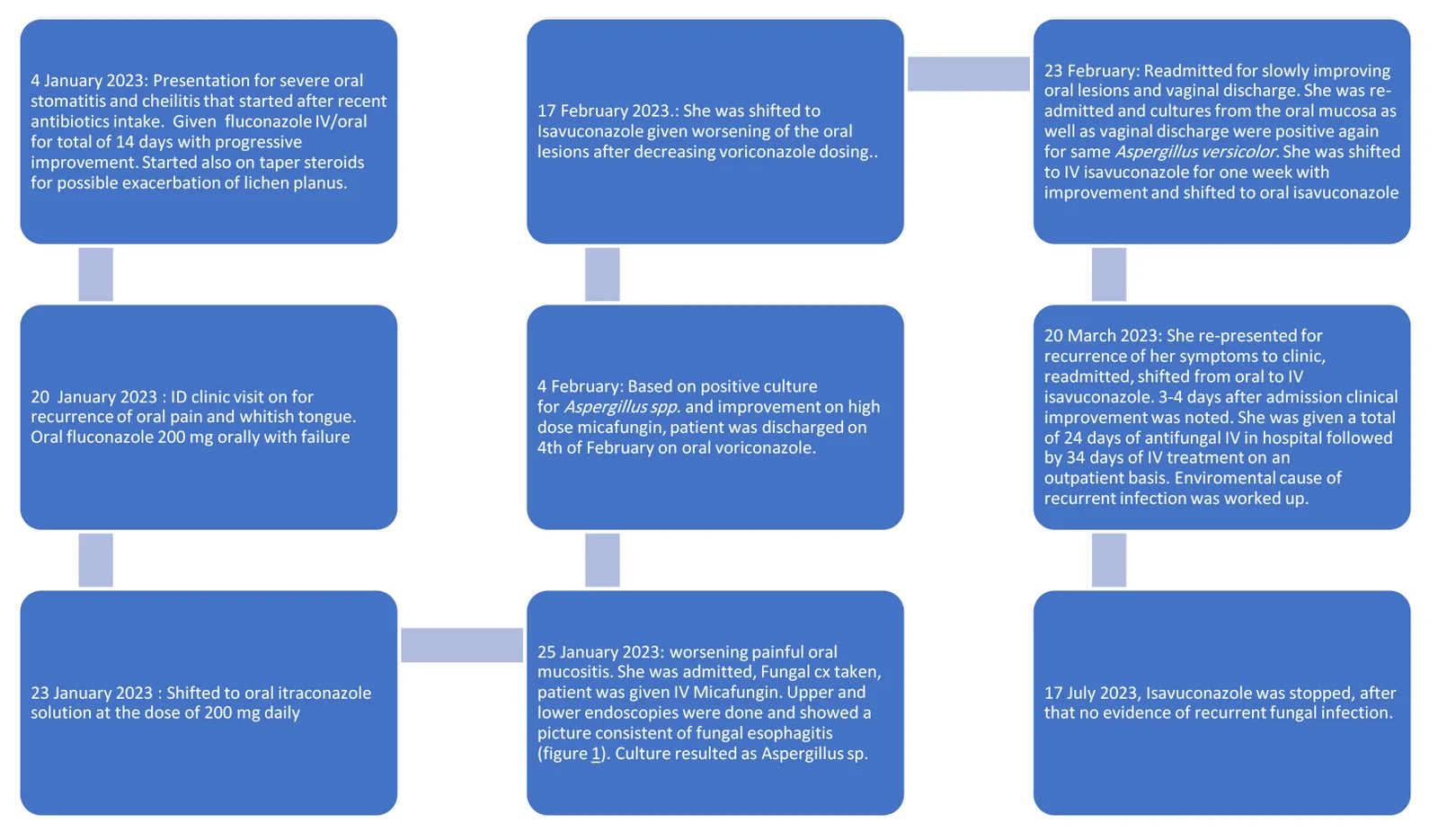
Timeline of events and treatment.

The case report was reviewed and approved by the institutional Publications Committee. As per institutional policy, ethical review and approval are not required for a single case report.

## Discussion

To our knowledge, this is the first reported case of invasive aspergillosis presenting as both stomatitis and vaginitis in a relatively immunocompetent patient, aside from recent short-term corticosteroid use for lichen planus. The initial clinical suspicion was directed toward *Candida* stomatitis, the more common etiology in such settings. Diagnosis of aspergillosis was made after a culture was obtained due to persistent lesions despite appropriate antifungal therapy targeting Candida. Invasive mold infections of the oral mucosa are rare in individuals without significant immunosuppression. While other *Aspergillus* species have been reported, to the best of our knowledge, this is the first documented case of *Aspergillus versicolor* causing oral mucositis in either immunocompromised or immunocompetent individuals. Patil et al. described one case of aspergillus oral infection causing extensive maxillary necrosis in an otherwise immunocompetent patient after tooth extraction [6]. Diagnosis was based on clinical, histopathology findings of hyphae as well as imaging results. Treatment was surgical sphenoidectomy and ethmoidectomy in addition to intravenous amphotericin B. A rare case of mandibular osteomyelitis due to aspergillosis in an immunocompetent adult and a different case of extension of aspergillosis into the oral cavity in a child have also been described [7, 8].

Good response to anti-mold therapy, like echinocandins and mold-active azoles, and repeated isolation of *Aspergillus versicolor* make the diagnosis of invasive aspergillus infection more likely. It is noteworthy that the patient had recurrent symptoms after shifting from IV to oral mold-active agent, mainly isavuconazole. She responded by re-challenging with the same IV antifungal despite its good oral bioavailability and absence of gastrointestinal problems that may alter the absorption of the drug. Her symptoms recurred every time she went home; therefore, potential environmental exposure to *Aspergillus* at home was considered and investigated. *Aspergillus versicolor*, identified morphologically by MALDI-TOF, was isolated from the air conditioning vent swab culture. Thereafter, deep cleaning and fumigation, including that of the air conditioning ducts, were performed.

Myoken et al. also described a potential environmental exposure to *Aspergillus* spp. using DNA amplification methods [4]. Three out of six *Aspergillus* infections were believed to be caused by the same single strain of *Aspergillus flavus*, and this was linked to a possible nosocomial outbreak of infection in the same hospital [4]. Despite the recurrence pattern of the infection, this patient was able to achieve remission of aspergillosis after prolonged antifungal therapy, with an attempt to reduce environmental exposure and control the underlying lichen planus disease using immunosuppressive drugs.

## Conclusion

Although candidiasis remains the most common cause of mucosal lesions, *Aspergillus* spp. should be considered in the differential diagnosis, particularly in patients with underlying mucosal barrier disruption, such as lichen planus. In this case, the isolation of *Aspergillus versicolor*, a relatively uncommon species within the *Aspergillus* genus, was an unexpected finding. This case highlights the importance of obtaining cultures from mucosal lesions prior to initiating antifungal therapy and underscores the need to consider potential environmental sources in patients with recurrent fungal infections.
